# Informed choice of pregnant women regarding noninvasive prenatal testing in Korea: a cross-sectional study

**DOI:** 10.4069/kjwhn.2022.09.10

**Published:** 2022-09-30

**Authors:** Hyunkyung Choi

**Affiliations:** College of Nursing and Research Institute of Nursing Science, Kyungpook National University, Daegu, Korea

**Keywords:** Cell-free DNA, Conflict, psychological, Counseling, Decision making, Prenatal care

## Abstract

**Purpose:**

This study explored the degree to which pregnant women in Korea made informed choices regarding noninvasive prenatal testing (NIPT) and investigated factors influencing whether they made informed choices.

**Methods:**

In total, 129 pregnant women in Korea participated in a web-based survey. Multidimensional measures of informed choice regarding NIPT and decisional conflict were used to measure participants’ levels of knowledge, attitudes, deliberation, uptake, and decisional conflict related to NIPT. Additional questions were asked about participants’ NIPT experiences and opinions.

**Results:**

All 129 pregnant women were recruited from an online community. Excluding those who expressed neutral attitudes toward NIPT, according to the definition of informed choice used in this study, only 91 made an informed choice (n=63, 69.2%) or an uninformed choice (n=28, 30.8%). Of the latter, 75.0% had insufficient knowledge, 39.3% made a value-inconsistent decision, and 14.3% did not deliberate sufficiently. No difference in decisional conflict was found between the two groups. A significant difference was found between the two groups in the reasons why NIPT was introduced or recommended (*p*=.021). Multiple logistic regression analysis showed that pregnant women who were knowledgeable (odds ratio [OR], 4.77; 95% confidence interval [CI], 2.17–10.47) and deliberated (OR, 0.74; 95% CI, 0.57–0.98) were significantly more likely to make an informed choice.

**Conclusion:**

The results of this study help healthcare providers, including nurses in maternity units, understand pregnant women’s experiences of NIPT. Counseling strategies are needed to improve pregnant women’s knowledge of NIPT and create an environment that promotes deliberation regarding this decision.

## Introduction

Noninvasive prenatal testing (NIPT) is a method to screen for chromosomal abnormalities in the fetus, such as Down syndrome, Patau syndrome, and Edward syndrome, using cell-free DNA fragments from the fetus that are circulating in a pregnant woman’s blood [1]. In particular, NIPT has been reported to have high sensitivity and specificity in assessing the risk of Down syndrome [2] and reduce the necessity of invasive prenatal testing, such as chronic villus biopsy and amniocentesis [3]. NIPT is possible from the 10th week of gestational age. Due to its safety for the fetus, the accuracy of its results, and the convenience of testing [4], NIPT has become commercially available in more than 60 countries, including South Korea (hereafter, Korea), since it was first introduced to clinical practice in Hong Kong in 2011 [5].

In 2019, the Korean Society of Maternal Fetal Medicine prepared medical guidelines for NIPT, with recommendations to inform and offer NIPT choice to all pregnant women, preferentially for women with high-risk pregnancies, to screen for trisomy 21, 18, and 13 and sex chromosome aneuploidy [6]. In Korea, high-risk pregnancies, including those in women with advanced maternal age (35 years or older), are steadily increasing [7], and it is expected that the use of NIPT will expand among these women.

Internationally, since NIPT has been introduced, various studies have examined knowledge and attitudes regarding NIPT among healthcare providers [8], pregnant women [9], women of childbearing age [10], and parents of children with Down syndrome [11]. Based on these studies, proactive discussions have been held on the ethical, legal, and social implications of NIPT and the role of healthcare providers [12]. In particular, the potential challenges and concerns about NIPT reported by healthcare providers, social science and humanities researchers, patient rights advocates, and religious group experts are as follows: proper consultations for pregnant women, pressure to undergo NIPT and elective abortion, discrimination against people with disabilities and reduction of social support, and making NIPT a routine prenatal test [4]. These studies emphasized informed decision-making and informed choice as the most important principles in the clinical practice of NIPT to promote women’s reproductive autonomy [13].

Informed choice, which is crucial for all treatments and medical tests, is based on the relevant knowledge, consistent with the values of the individual who makes decisions, and behaviorally practiced accordingly [14]. Marteau et al. [14] presented knowledge, attitudes, and uptake as three concepts that are important for making an informed choice based on the theory of planned behavior, and developed an instrument measuring informed choice during prenatal testing based on the multifaceted relationship among these variables. In addition, with an emphasis on the importance of deliberation before making a certain decision, Lewis et al. [15] added the concept of deliberation to the instrument developed by Marteau et al. [14] and developed a multidimensional instrument measuring informed choice in NIPT situations based on the relationship among these variables. According to the instrument, informed choice for a specific test involves (1) accepting the test with a positive attitude and deliberation with sufficient relevant knowledge about the test or (2) declining the test due to a negative attitude toward the test, despite having sufficient relevant knowledge about the test and having deliberated. In other words, a woman makes an uninformed choice with a lack of relevant knowledge and/or when her attitude is not reflected in her behavior such as declining the test with a positive attitude or accepting the test with a negative attitude. Therefore, in order to promote informed choice for NIPT, it is crucial for healthcare providers, who have sufficient knowledge of NIPT, to provide accurate information about the risks, benefits, procedures, and costs of NIPT with a value-neutral attitude and to support pregnant women to make decisions consistent with their values after sufficient deliberation. Meanwhile, various efforts have been made to aid pregnant women’s decision-making to promote informed choice in prenatal testing, including NIPT, and it was reported that these interventions reduced decisional conflict and promoted informed choice [16].

Decisional conflict refers to the uncertainty experienced in deciding upon a certain behavior [17]. It is more likely to occur when making a risky or uncertain decision, when a compromise is made between values during the process of decision-making, and when making a decision for which one expects regret regarding the positive aspects of a refused option [17]. In particular, uncertainty increases when an individual feels that there is a lack of information about alternatives, benefits, and risks, personal values are unclear, and there is no support to make a certain decision or pressure to make a decision [18]. Since prenatal testing, particularly NIPT, is accompanied by uncertainty about the fetal condition, decisional conflict may occur [19]. According to O’Connor [18], this uncertainty can be reduced by providing information about alternatives, benefits, risks, and side effects, helping individuals to clarify the values that they consider important and supporting the deliberation process.

Healthcare providers, including nurses who provide prenatal management, should help pregnant women make autonomous informed choices without decisional conflict. In particular, nurses are in an optimal position to perform this role [20]. In addition, the role of nurses as advocates is now increasingly emphasized [21]. Although a study attempted to examine the proper clinical applications and nursing implications of NIPT at the initial stage of the introduction of NIPT in Korea [22], since then, only one study has examined healthcare providers’ attitudes toward NIPT and its implementation [23]. To our best knowledge, there are yet no studies on pregnant women’s NIPT-related experiences in Korea.

Meanwhile, web-based data collection has been used in various academic fields since it was introduced in the late 1990s, and its proactive use has been expected in epidemiology, which studies various factors affecting health and disease within specific populations [24]. Since internet use in Korea reached 96.5% in 2020 [25], the proper use of carefully designed web-based questionnaires can complement or serve as an alternative to traditional data collection [24]. In fact, in response to limitations of face-to-face contact due to coronavirus disease 2019 (COVID-19), many health-related studies have been conducted using web-based methodologies. In particular, since social desirability bias can affect pregnant women’s responses to questions about attitudes toward prenatal testing [24], a web-based survey that can elicit honest answers is appropriate.

Therefore, by examining pregnant women’s informed choice of whether to undergo NIPT, factors influencing their informed choice, and NIPT-related experiences through a web-based questionnaire, this study aimed to provide basic data to help healthcare providers, including nurses, establish counseling strategies for NIPT that can promote informed choice by pregnant women. The detailed goals are as follows: (1) to identify the general and obstetric characteristics of pregnant women; (2) to examine the characteristics of pregnant women’s NIPT-related experiences; (3) to identify the scores for main variables and the degree to which pregnant women made informed choices regarding NIPT; (4) to examine the level of informed choice according to pregnant women’s general and obstetric characteristics; (5) to examine differences in the main variables according to informed choice; and (6) to identify factors associated with informed choice.

## Methods

Ethics statement: This study was approved by the Institutional Review Board of Kyungpook National University (2022-0003). Informed consent was obtained from the participants.

### Study design

This correlational study using a cross-sectional survey was conducted to investigate pregnant women’s level of informed choice of whether to undergo NIPT and factors influencing informed choice. The reporting of this study followed the STROBE (STrengthening the Reporting of OBservational studies in Epidemiology) reporting guidelines (https://www.strobe-statement.org/).

### Participants

The participants of this study were pregnant women who had requested NIPT or had been introduced to or recommended NIPT by their healthcare provider. The other inclusion criteria were pregnant women aged 19 years or older living in Korea who were at a gestational age of 10 weeks or higher during the study period and voluntarily agreed to participate in the study after understanding its purpose. Since this study examined the level of knowledge about NIPT, it excluded foreigners, marriage migrant women, and pregnant women with difficulties in reading and understanding Korean. The number of participants required for analysis was calculated to be 121 using G*Power 3.1.9.7, with settings of a two-tailed test, significance level of .05, power of .95, and an odds ratio (OR) of 2.37 [26] in logistic regression analysis. Among 150 responses to the questionnaire, excluding 21 cases with insincere or incomplete responses, 129 cases were used for the final analysis, and the number of participants satisfied the minimum sample size.

### Instruments

#### General and obstetric characteristics

The general characteristics include age, educational level, financial status, employment status, religion, and individual experience with people with chromosomal disabilities. The obstetric characteristics consisted of gestational age (weeks), method of pregnancy, parity, reason for being introduced to or recommended NIPT, and the place of prenatal care.

#### Informed choice of whether to undergo noninvasive prenatal testing

The multidimensional measure of informed choice of whether to undergo NIPT (MMIC-NIPT) developed by Lewis et al. [15] comprises a total of 24 items on knowledge (12 items), attitudes (five items), deliberation (six items), and uptake (one item). The items on knowledge include participants’ knowledge of NIPT, Down syndrome, and others, which is classified as good or poor based on a cutoff of 9 out of 12 points. The items on attitudes ask about attitudes toward conducting NIPT, and responses are classified into positive (0–6 points), neutral (7–13 points), and negative (14–20 points). Participants with a neutral attitude are excluded when classifying them into groups according to the level of informed choice. The items on deliberation relate to whether participants deliberated about alternative evaluations, results, and the advantages and disadvantages of NIPT, and a score of 12 points or lower out of a possible score of 24 is considered to indicate that a participant has sufficiently deliberated. Lastly, the item on uptake asks about choices regarding NIPT.

In this study, after receiving approval from the developer of the original instrument, the instrument was translated and reviewed, back-translated, and reviewed by experts, and a preliminary study was conducted following the standard procedure [27] presented by the World Health Organization. Twenty-three modified items that fit the domestic situation were finally used for measurements. Two people whose native languages were Korean and who were proficient in English translated the instrument into Korean, and two bilingual experts compared the original and translated instruments and reviewed inappropriate expressions or conflicts of meaning. The back-translation was conducted by a person whose native language was English and who was proficient in Korean, but had no knowledge of the instrument. A native speaker of English confirmed the consistency of meaning of 24 items in the original and back-translated instruments. The expert group, which consisted of three professors in nursing and two nurses with 9 or more years of work experience in the delivery room, confirmed the content validity index of the translated instrument. When the content validity of each item is 0.78 or higher and the content validity of the entire instrument is 0.90 or higher, it is considered good [28]. In this study, the content validity of the entire instrument was 0.96, except for one item with a content validity of less than 0.8. Using the 23 modified items after the expert content validity review, a preliminary study was conducted on 14 pregnant women at a gestational age of 10 weeks or higher who had been introduced to or recommended NIPT. Based on the results of the preliminary study, the expressions in the multiple-choice responses of one item measuring knowledge were partially modified.

The final modified items used in this study consisted of 23 items on knowledge (11 items), attitudes (five items), deliberation (six items), and uptake (one item). For items measuring knowledge the answer “do not know” was considered incorrect, and the highest possible summed score was 11 points. A higher score indicated higher knowledge about NIPT. In the study of Lewis et al. [15], a good level of knowledge was defined as a score of 9 points or higher, whereas this study set a cutoff score of 8 points or higher (corresponding to approximately 70%) out of 11 after the expert group discussion. Scores for attitudes and deliberation were classified according to the criteria of the original instrument. The items on attitudes measured how the pregnant women felt about NIPT using a 5-point Likert scale (e.g., 0, beneficial to 4, harmful). A lower summed score (possible range, 0–20) indicated a positive attitude toward NIPT. The items on deliberation were measured using a 5-point Likert scale (0, very to 4, not at all), and a lower summed score (possible range, 0–24) indicated a higher level of deliberation on NIPT. Finally, the item on uptake asked whether NIPT was conducted. Cronbach’s α of reliability in the study of Lewis et al. [15] was 0.69 for knowledge, 0.94 for attitudes, and 0.84 for deliberation, and the corresponding values in this study were 0.70, 0.87, and 0.86, respectively. When analyzing pregnant women’s level of informed choice according to their scores of knowledge, attitudes, deliberation, and uptake, those who reported neutral attitudes are excluded based on the concept of value consistency [15]. Informed choice included participants who (1) agreed to undergo NIPT after sufficient deliberation (12 points or lower) with good knowledge (8 points or higher) and a positive attitude (6 points or lower) or (2) declined NIPT with a negative attitude (14 points or higher) despite having good knowledge and sufficient deliberation. Uninformed choice included cases of having insufficient knowledge, not deliberating, and/or value inconsistency such as having a positive attitude but declining NIPT or having a negative attitude but accepting NIPT.

#### Decisional conflict on noninvasive prenatal testing

In this study, the decisional conflict scale (DCS) developed by O’Connor [17] and translated into Korean by Yun et al. [29] was used. The original scale was developed for the development and evaluation of ancillary interventions in order to reduce uncertainty in health-related decision-making that individuals feel in various clinical environments, and it has been used in several previous studies on NIPT [19,30]. The DCS consists of five sub-factors (uncertainty, informed, values clarity, support, and effective decision), with 16 items in total. Each item is measured using a 5-point Likert scale (0, strongly agree to 4, strongly disagree) and the total score is divided by 16 and multiplied by 25 such that 100 points constitute a perfect score. A lower score indicates a lower level of decisional conflict on NIPT, and decisional conflict is considered to occur when the score is 37.5 points or higher. The Cronbach’s α of reliability was 0.78 when the scale was developed, 0.90 in the study of Yun et al. [29], and 0.94 in this study.

#### Noninvasive prenatal testing-related experiences

Referring to previous studies [19,30], an 11-item questionnaire was prepared about experiences of NIPT from various perspectives (e.g., satisfaction with explanations about NIPT, reasons for accepting or declining NIPT) and opinions on when NIPT should be conducted. The level of satisfaction with the explanations on NIPT was evaluated through five items on the degree of difficulty, quantity, delivery method, content, and usefulness of the information provided by healthcare providers. The other items dealt with the most important reason for accepting NIPT (one item), the most important factor in the choice of whether to undergo NIPT (one item), the most important reason to decline NIPT (one item), the person with the most influence on the choice of whether to undergo NIPT (one item), satisfaction with choice of whether to undergo NIPT (two items), and opinions on when NIPT should be conducted (one item).

### Data collection

After receiving approval from the institutional review board of the researcher’s affiliated university, data were collected in January 2022. An online format was used due to COVID-19-related restrictions on face-to-face contact and to elicit honest responses. A recruitment notice including the research purpose and methods was posted on a domestic online community for expecting mothers, and participants who were interested in the study could directly access the questionnaire with a URL or QR code. Participants were able to start the questionnaire only after agreeing to participate in the study. If participants did not agree to participate in the study or the exclusion criteria applied to them, they were not allowed to start the questionnaire. If participants met the inclusion criteria, they could respond to the questionnaire after providing online consent. The survey took 10-–15 minutes, and the participants received a small gift (worth 4 US dollars).

### Data analysis

The collected data were analyzed using IBM SPSS ver. 25.0 (IBM Corp., Armonk, NY, USA). Descriptive statistics were used to identify the general and obstetric characteristics of pregnant women, pregnant women’s experiences regarding NIPT, the level of the main variables, and informed choice according to the participant characteristics. The chi-square test or the Fisher exact test was conducted to identify the level of informed choice according to the general and obstetric characteristics of pregnant women. The Mann-Whitney U-test was conducted to identify differences in informed choice according to the main variables. To identify factors influencing informed choice, univariate logistic regression analysis and multiple logistic regression analysis were conducted.

## Results

### General and obstetric characteristics of pregnant women

The mean age of the 129 pregnant women was 34.08±3.47 years (range, 25-–44 years), and 48.1% were 35 years of age or older. Most (89.1%) had an educational level of university or higher, and 88.4% reported a financial status of middle or higher. Housewives accounted for 59.7% of the participants, and 65.1% were not religious. Most of the pregnant women (81.4%) did not have individual experience with people with chromosomal disabilities. The gestational age ranged from 10 weeks and 2 days to 38 weeks and 5 days, and 55.8% and 35.7% were in the second trimester and third trimester, respectively. Ninety-three percent of participants had a spontaneous pregnancy, and 69.0% were nulliparous. The most common single reason for being introduced to or recommended NIPT by healthcare providers was the pregnant woman’s wishes (42.6%), while 57.4% were introduced to or recommended NIPT for a variety of reasons related to a high-risk pregnancy, including advanced maternal age (35 years or older), high-risk status based on standard prenatal blood tests, abnormal ultrasound results, and chromosomal abnormalities in a past pregnancy. The participants most commonly received prenatal care at women’s hospitals (45.7%) and tertiary or general hospitals (28.7%) (Table 1).

### Pregnant women’s experiences of noninvasive prenatal testing

#### Satisfaction with noninvasive prenatal testing explanations

The NIPT information provided by healthcare providers was easy or somewhat easy to understand for 64.4% of pregnant women, while 75.2% responded that the amount of NIPT information was appropriate. Furthermore, 64.3% of pregnant women responded that the NIPT information was presented in a way that they could understand and 69.0% acknowledged that the NIPT information covered topics they wanted to know about. The percentage of pregnant women who responded that the NIPT information assisted in their decision-making was 78.3% (Table 2).

#### Reasons for accepting or declining noninvasive prenatal testing

The most important reason for accepting NIPT was making sure that their child did not have a chromosomal abnormality (61.9%), followed by getting as much information about their baby as possible (14.4%), getting help on the decision of whether to continue the pregnancy (12.4%), and preparing and planning the delivery of a baby with a chromosomal abnormality (10.3%). The most important factor in choosing NIPT was safety for the fetus (42.3%), followed by the possibility of early testing (27.8%), accuracy of results (25.8%), and convenience of the test (4.1%). The most important reason for declining NIPT was its high cost (50.0%), followed by the probability of false-negative and false-positive results (40.6%) (Table 2).

#### The person with the greatest influence on the noninvasive prenatal testing choice and satisfaction with the choice of whether to undergo noninvasive prenatal testing

As the person with the greatest influence on the NIPT choice, pregnant women noted themselves (42.6%), followed by healthcare providers (34.1%), spouses (16.3%), and family members and friends (7.0%). The majority of pregnant women (77.6%) were satisfied with their choice of whether to undergo NIPT, while 20.2% and 2.3% reported neutral feelings and dissatisfaction, respectively (Table 2).

#### Opinions on when noninvasive prenatal testing should be conducted

According to the results of a multiple-choice question, the most frequent response regarding when NIPT should be conducted was “when pregnant women wish to be tested” (n=84) followed by “for pregnant women over age 35” (n=69), “when pregnant women are at high risk on standard screening tests” (n=66), “when abnormal findings are found on fetal ultrasonography” (n=50), “for pregnant women who have had chromosomal abnormalities in the past” (n=41), “for all pregnant women” (n=39), and “if either parent has a chromosomal abnormality” (n=35) (Table 2).

### Values of main variables and frequency of making an informed choice regarding noninvasive prenatal testing

The score for knowledge on NIPT of all pregnant women was 7.87±2.62 points on average, and 64.3% showed good knowledge (≥8 points). The average score for attitudes toward NIPT was 5.53±3.84 points (positive, 68.2%; neutral, 29.5%; and negative, 2.3%). The average score for deliberation on NIPT was 6.90±4.28 points, and 86.8% ( ≤12 points ) reported sufficient deliberation. The percentage of pregnant women who accepted NIPT was 75.2%, while 24.8% declined NIPT. The average score of decisional conflict was 26.88±12.96 points, and 18.6% (n=24) noted having decisional conflict related to NIPT (≥37.5) (Table 3).

With the exclusion of 38 pregnant women who reported neutral attitudes, the frequency of informed choice was calculated for 91 pregnant women. Among them, 69.2% (n=63) made an informed choice, i.e., these women accepted NIPT with good knowledge, positive attitudes, and sufficient deliberation. There were no pregnant women who declined NIPT with good knowledge and sufficient deliberation, but negative attitudes. However, 30.8% of pregnant women (n=28) made an uninformed choice. Among them, 75.0% (n=21) made a decision with insufficient knowledge, 14.3% (n=4) did not deliberate, and 39.3% (n=11) made a value-inconsistent choice (Table 4).

### The frequency of making an informed choice regarding noninvasive prenatal testing according to pregnant women’s general and obstetric characteristics

There was no significant difference in informed choice according to the general characteristics of pregnant women. However, among the obstetric characteristics, significant difference between two groups was found in the reason for being introduced to or recommended NIPT (*p*=.021). Specifically, 61.9% of pregnant women themselves requested information about NIPT in the informed choice group, whereas in the uninformed choice group, 64.3% of pregnant women were introduced to or recommended NIPT due to factors indicative of a high-risk pregnancy, such as advanced maternal age (35 years or older) and high-risk findings on standard prenatal blood tests (Table 1 ).

### Differences in main variables according to whether participants made an informed choice regarding noninvasive prenatal testing

The pregnant women who made an informed choice had significantly higher average knowledge scores than the pregnant women who made an uninformed choice (*p*<.001) and deliberated more (*p*=.019). There was no significant difference in the total score for decisional conflict between the two groups. However, the pregnant women who made an uninformed choice were significantly less likely to consider their decisions to be effective than the pregnant women who made an informed choice (*p*=.013) (Table 3).

### Factors influencing whether pregnant women made an informed choice regarding noninvasive prenatal testing

According to the results of univariate logistic regression on the pregnant women’s general characteristics (age, educational level, financial status, job, religion, and individual experience with people with chromosomal disability), obstetric characteristics (method of pregnancy, parity, reason for being introduced to or recommended NIPT, and place of prenatal care), knowledge, attitudes, deliberation, and decisional conflict, the pregnant women who had prenatal care at tertiary or general hospitals showed a 3.64 times (95% confidence interval [CI], 1.05–12.55) higher likelihood of making an informed choice than the pregnant women who had prenatal testing at obstetrical and gynecologic clinics. As the level of knowledge increased, the likelihood of making an informed choice was 3.38 times (95% CI, 2.02–5.66) greater. However, the likelihood of making an informed choice when pregnant women were introduced to or recommended NIPT for reasons related to a high-risk pregnancy was approximately 2/3 lower (OR, 0.34; 95% CI, 0.14–0.86) than women who requested NIPT. As the attitude of pregnant women was more negative, the likelihood of making an informed choice was 1/5 lower (OR, 0.80; 95% CI, 0.66–0.97). Also, the less deliberate the pregnant women were, the likelihood of making an informed choice was approximately 1/5 lower (OR, 0.79; 95% CI, 0.67–0.093).

The results of multiple logistic regression, where all variables were entered simultaneously, showed that the regression model yielded significant results (χ^2^=63.35, *p*<.001). The Hosmer-Lemeshow test confirmed that the model was suitable (*p*=.753). The explanatory power was 50.1% according to Cox and Snell’s coefficient of determination (R^2^) and 70.7% according to Nagelkerke’s coefficient of determination (R2). A higher level of knowledge was associated with a 4.77 times higher likelihood (95% CI, 2.17–10.47) of making an informed choice, whereas the likelihood of making an informed choice was approximately 1/4 lower (OR, 0.74; 95% CI, 0.57–0.98) when the level of deliberation was insufficient (Table 5).

## Discussion

In Korea, as high-risk pregnancies become increasingly common and the incidence of hereditary diseases increases with the aging of pregnant women, interest in NIPT guaranteeing the safety of the fetus among pregnant women is high. This web-based cross-sectional study—the first of its kind in Korea, to our best knowledge—was conducted to investigate the level of informed choice among pregnant women regarding whether to undergo NIPT and to identify factors influencing informed choice.

In this study, 64.3% of pregnant women showed good knowledge of NIPT, which is substantially lower than the findings of 95% in a study on pregnant women in the United Kingdom [15] and 88.3% in a study on pregnant women in Australia [30], which measured the level of knowledge using the same instrument. In addition, 68.2% of pregnant women showed positive attitudes toward NIPT in this study, which is also lower than the results of 88% and 80.9% in the United Kingdom and Australia, respectively [15,30]. Although our study identified 86.8% of pregnant women reporting sufficient deliberation on NIPT, nevertheless this is a lower percentage than that of pregnant women (92%) in the United Kingdom. Altogether, 69.2% of pregnant women made an informed choice in this study, which is also lower than the percentage of 89% reported in a study on pregnant women in the United Kingdom [15]. This result may be due to differences in participants. A previous international study [15] was conducted on pregnant women who received moderate or high-risk results in Down syndrome screening and were provided written materials on NIPT and an individual pre-consultation from a midwife before choosing NIPT. However, in this study, 42.6% of pregnant women were provided information on NIPT from healthcare providers because they wished to. Thus, it can be inferred that the characteristics of this study population are somewhat different from those who received a consultation conducted only for women with high-risk pregnancies.

Furthermore, in this study, 75% of pregnant women who made an uninformed choice made decisions without having sufficient knowledge of NIPT; this proportion is very high compared to the results (45.8%) of a previous study on pregnant women in the United Kingdom [31]. Value inconsistency between attitudes on NIPT and uptake occurred among 39.3% of pregnant women in this study, which is also higher than the percentage of 13.2% in the previous study [31]. A likely explanation for this discrepancy is that in the United Kingdom study, educated midwives provided 30-minute consultations on NIPT for pregnant women with high-risk pregnancies for Down syndrome; thus, possibly fewer women with insufficient knowledge. Another study on attitudes toward NIPT among Korean clinicians, however, found that 70.9% spent 5 minutes or less conducting consultation of prenatal testing [23], and the actual outpatient treatment time in most departments, including obstetrics, was shorter than the treatment time that would satisfy patients [32]. In addition, the fact that pregnant women in the United Kingdom study [31] could choose NIPT without additional cost might explain the high level of value consistency that they reported. Even if pregnant women have positive attitudes, they may not be able to take action due to various circumstantial factors. For example, some pregnant women may prefer to have an invasive diagnostic test right away, rather than waiting 7 to 10 days for the NIPT results [31]. Therefore, in order to comprehensively understand pregnant women’s informed choice regarding NIPT, replication studies reflecting these aspects of pregnant women’s situations are necessary.

According to O’Connor [18], if the score for decisional conflict was 25 points or less, the subjects were considered to have made clinical decisions without decisional conflict, whereas a score of 37.5 points or higher indicated that the subjects experienced decisional conflict, such as delaying decision-making or feeling uncertainty in taking actions. Although 72.5% of pregnant women in this study had scores of 25 points or less and accepted or declined NIPT without decisional conflict, 18.6% had scores of 37.5 points or higher, which means that they experienced decisional conflict. Decisional conflict occurs as a result of difficulties inherent in the type of decisions, but several cognitive, emotional, and social factors can further exacerbate decisional conflict [18]. Knowledge about NIPT can act as a cognitive factor, and sufficient knowledge is essential in informed choice. Various attempts have been made internationally in order to provide sufficient knowledge to pregnant women during consultations. Dane et al. [33] investigated NIPT-related items (the accuracy, advantages, and disadvantages of NIPT compared to other tests) that pregnant women considered most important to make an informed choice, and this approach can be used effectively if the consultation should be conducted in a limited time. This is worthy to consider as this study found only 64.3% of pregnant women responded that the information on NIPT was presented in a way that they could understand, and only 77.6% of pregnant women were satisfied with decision-making on NIPT. Therefore, it is necessary to examine what information pregnant women value and how they prefer that information to be delivered.

Among the general and obstetric characteristics in this study, the likelihood of making an informed choice was low when pregnant women were introduced to or recommended NIPT by healthcare providers for reasons related to a high-risk pregnancy, whereas high when they received prenatal care at tertiary or general hospitals. Although the interpretation of these findings is limited since there are no previous studies using the same variables as this study, healthcare providers generally recommend NIPT in cases of advanced maternal age (35 years or older), high-risk findings from standard prenatal blood tests, and abnormal results from ultrasound [23]. In these cases, pregnant women may accept NIPT without sufficient deliberation to assure themselves of the well-being of their fetus even if it is not consistent with their own attitudes toward NIPT. This can be understood as aligning with the fact that 61.9% of pregnant women in this study accepted NIPT to make sure their child did not have a chromosomal abnormality. Meanwhile, since tertiary or general hospitals deal with high-risk pregnancies more often than obstetrical/gynecologic clinics, the education and preparation of healthcare providers on NIPT consultations might be more systematic, which likely had a positive influence on the informed choice of pregnant women. However, in this study, age, educational level, and religion, which were confirmed as influencing factors in previous studies [26,31,34,35], did not show significant influences on pregnant women’s informed choice. Therefore, in addition to the characteristics investigated in this study, replication studies including health literacy [36], which has been previously reported as a significant influencing factor, are required.

In this study, the likelihood of an informed choice among pregnant women increased with higher knowledge but decreased with insufficient deliberation. In order to increase the NIPT-related knowledge of pregnant women and help them make an informed choice, it is necessary to establish counseling strategies that can provide accurate knowledge on NIPT effectively in a limited clinical environment. The results of this study could be fairly predictable, since sufficient knowledge and deliberation were reflected when classifying whether women made an informed choice. Nonetheless, this study was significant in that the influence of knowledge and deliberation was confirmed while controlling for other general and obstetric characteristics. Beulen et al. [26] reported that the use of a web-based multimedia decision aid increased pregnant women’s level of knowledge about NIPT and was effective for promoting informed decision-making. Therefore, the proper use of decision aids can be considered when establishing NIPT counseling strategies. Moreover, the results of this study show the importance of preparing an environment where pregnant women can deliberate on NIPT sufficiently. In a previous international study [37], 66.9% of pregnant women wished to have the test on the same day when the NIPT-related consultation was conducted, whereas 70.8% of healthcare providers responded that the next visit was appropriate. Considering the differences in perspectives on the timing of testing, counseling strategies that help pregnant women sufficiently deliberate will be required.

The person with the greatest influence on NIPT choice in this study was pregnant women themselves (42.6%), followed by healthcare providers (34.1%). In comparison, a study on pregnant women in Canada [38] reported more than 80% of pregnant women responded that healthcare providers had an influence on their NIPT choice to some extent, and 74% responded that disagreement with their spouse did not have a significant influence on NIPT choice. In other words, although pregnant women themselves are the most important influence on accepting or declining NIPT, healthcare providers can have a significant influence on pregnant women’s decisions. In this study, 10.3% and 12.4% of pregnant women accepted NIPT to prepare and plan the delivery of a baby with a chromosomal abnormality and to get help with the decision of whether to continue the pregnancy, respectively. Hence, in order for pregnant women to have autonomy in accepting or declining NIPT, healthcare providers should be able to support an informed choice by providing necessary information (e.g., balanced information on people with Down syndrome [39]) through nondirective counseling.

The pregnant women in this study expressed the opinion that all pregnant women, including those with high-risk factors, who wish to be tested should be able to receive NIPT. However, 50% of pregnant women in this study who declined NIPT did so due to high costs. A previous international study also suggested that test cost is a factor influencing NIPT choice, reporting that the level of NIPT acceptance among pregnant women living in regions with low socioeconomic levels was significantly lower than among women from other regions [40]. The cost of NIPT varies from country to country [13]. The Health Insurance Review and Assessment Service announced that average cost of NIPT in Korea was about 600,000 Korean Won (approximately 450 US dollars), and pregnant women have to cover this cost since the test is not covered by insurance [41]. Some European countries provide political support for NIPT; for instance, Belgium and the Netherlands provide NIPT to all pregnant women and compensate part or all of the cost [42]. NIPT should be accessible to all pregnant women wishing to be tested to ensure women’s reproductive autonomy. However, there are also substantial ethical concerns about the routinization of NIPT as prenatal testing by policy or social pressure, as it could lead to elective terminations of pregnancies [43]. Recently in Korea, the abortion law has been amended and legal restrictions on abortion under the criminal law disappeared [44]. Therefore, an informed choice of whether to undergo NIPT and deliberation in that process became even more important. In order to help pregnant women make an informed choice, it is necessary to establish appropriate counseling strategies and simultaneously hold proactive discussions on the ethical and social impacts of NIPT.

This web-based cross-sectional study investigated pregnant women’s level of informed choice regarding NIPT, as well as factors influencing their likelihood of making an informed choice. However, caution is needed when making causal inferences or generalizing the findings to all pregnant women. This study confirmed that knowledge related to NIPT and deliberation were important factors associated with making an informed choice. Pregnant women’s knowledge about NIPT may be affected by various factors, such as NIPT experiences in previous pregnancies, but not all possible factors were considered in this study. Another limitation is that when a pregnant woman has sufficient knowledge and deliberates with a positive attitude, but declines NIPT due to high cost, that decision cannot be distinguishable from an uninformed choice. Nonetheless, this exploratory study reflects the first attempt to explore the NIPT experiences of pregnant women in Korea, where survey studies on NIPT in pregnant women are lacking. This study makes a significant contribution by elucidating pregnant women’s experiences with NIPT and presenting basic data that will help prepare counseling strategies to promote informed choices by pregnant women regarding whether to undergo NIPT in the future.

This study found a difference in the level of informed choice of pregnant women according to the reasons for being introduced to or recommended NIPT, and higher knowledge was associated with a higher likelihood of making an informed choice-NIPT. Based on these results, this study suggests the need to prepare counseling strategies on NIPT to enhance pregnant women’s knowledge, as well as considering measures to create an environment suitable for deliberation within the limitations of the clinical setting. Furthermore, since factors such as the educational level and religion of pregnant women [35] and the experience of prenatal testing for Down syndrome [31] have been identified as factors influencing informed choice in previous international studies, replication studies with expanded samples of participants are suggested.

## Figures and Tables

**Table 1.** Informed choice by general and obstetric characteristics (N=129)

Characteristics	Categories	n (%)			χ^2^(*p*)
		Total (N=129)	Informed choice (n=63)	Uninformed choice (n=28)	
*General characteristics*					
Age (year)	≤34	67 (51.9)	41 (65.1)	15 (53.6)	1.09 (.298)
	≥35	62 (48.1)	22 (34.9)	13 (46.4)	
Educational level	≤College	14 (10.9)	4 (6.3)	4 (14.3)	1.52^†^ (.245)
	≥University	115 (89.1)	59 (93.7)	24 (85.7)	
Financial status	High/middle-high	49 (38.0)	24 (38.1)	11 (39.3)	0.01 (.914)
	Middle/middle-low	80 (62.0)	39 (61.9)	17 (60.7)	
Job	No	77 (59.7)	37 (58.7)	16 (57.1)	0.02 (.887)
	Yes	52 (40.3)	26 (41.3)	12 (42.9)	
Religion	No	84 (65.1)	40 (63.5)	19 (67.9)	0.16 (.687)
	Yes	45 (34.9)	23 (36.5)	9 (32.1)	
Individual experience with people with chromosomal disability	No	105 (81.4)	47 (74.6)	24 (85.7)	1.40^†^ (.284)
	Yes	24 (18.6)	16 (25.4)	4 (14.3)	
*Obstetrical characteristics*					
Parity	Nulliparous	89 (69.0)	45 (71.4)	18 (64.3)	0.46 (.496)
	Parous	40 (31.0)	18 (28.6)	10 (35.7)	
Reason for introducing NIPT	Wanted	55 (42.6)	39 (61.9)	10 (35.7)	5.35 (.021)
	Reasons related to	74 (57.4)	24 (38.1)	18 (64.3)	
	a high-risk pregnancy				
Place of prenatal care	Tertiary or general hospital	37 (28.7)	24 (38.1)	6 (21.4)	4.44 (.108)
	Women’s hospital	59 (45.7)	28 (44.4)	12 (42.9)	
	Obstetrical/gynecologic clinic	33 (25.6)	11 (17.5)	10 (35.7)	

NIPT: Noninvasive prenatal testing.

† Fisher exact test.

**Table 2.** Experiences of pregnant women related to NIPT (N=129)

Questions, reasons, or opinions	Categories	n (%) or n only
*(1) Satisfaction with NIPT explanation*		
How easy was the NIPT information to understand?	Easy	22 (17.1)
	Somewhat easy	61 (47.3)
	Somewhat hard	44 (34.1)
	Hard	2 (1.6)
The amount of NIPT information	Too much	17 (13.2)
	The right amount	97 (75.2)
	Too little	15 (11.6)
The NIPT information was presented in a way that I could understand	Strongly agree	15 (11.6)
	Agree	68 (52.7)
	Neutral	40 (31.0)
	Disagree	5 (3.9)
	Strongly disagree	1 (0.8)
The NIPT information covered things I wanted to know	Strongly agree	16 (12.4)
	Agree	73 (56.6)
	Neutral	34 (26.4)
	Disagree	6 (4.7)
The NIPT information assisted in my decision-making	Strongly agree	20 (15.5)
	Agree	81 (62.8)
	Neutral	25 (19.4)
	Disagree	2 (1.6)
	Strongly disagree	1 (0.8)
*(2) Reasons for accepting or declining NIPT*		
The most important reason for accepting NIPT (n=97)	To prepare and plan the delivery of a baby with a chromosomal abnormality	10 (10.3)
	To get help with decision of whether to continue the pregnancy	12 (12.4)
	To make sure my child does not have a chromosomal abnormality	60 (61.9)
	To get as much information about my baby as possible	14 (14.4)
	Because the test is not at all dangerous to the baby	1 (1.0)
The most important factor in choosing NIPT (n=97)	Safety for fetus	41 (42.3)
	Possibility of early testing	27 (27.8)
	Accuracy of the result	25 (25.8)
	Convenience of the test	4 (4.1)
The most important reason for declining NIPT (n=32)	High cost	16 (50.0)
	Religious belief	1 (3.0)
	Possibility of false-negative and false-positive results	13 (40.6)
	Others	2 (6.3)
	Strong will to give birth (n=1)	
	Experience of giving birth to the first child without any issue (n=1)	
*(3-1) The person with the greatest influence on NIPT choice*		
	Myself	55 (42.6)
	Spouse	21 (16.3)
	Healthcare provider	44 (34.1)
	Family and friend	9 (7.0)
*(3-2) Satisfaction with choice of whether to undergo NIPT*		
	Strongly agree	18 (14.0)
	Agree	82 (63.6)
	Neutral	26 (20.2)
	Disagree	3 (2.3)
*(4) Opinions on when NIPT should be conducted (multiple choice)*		
	Pregnant women wishing to be tested	84
	Pregnant women over 35 years of age	69
	Pregnant women who are at high risk on standard screening tests	66
	Pregnant women with abnormal findings on fetal ultrasonography	50
	Pregnant women who have had chromosomal abnormalities in the past	41
	All women	39
	If either parent has a chromosomal abnormality	35

NIPT: Noninvasive prenatal testing.

**Table 3.** Level of the main variables and by informed choice (N=129)

Variable	Min	Max	Possible range	Mean±SD			U^‡^	*p*
				Total (N=129)	Informed choice (n=63)	Uninformed choice (n=28)		
MMIC-NIPT								
Knowledge	0	11.0	0–11	7.87±2.62	9.59±1.07	6.86±2.17	216.5	<.001
Good (≥8), 64.3%								
Poor (<8), 35.7%								
Attitude	0	15.0	0–20	5.53±3.84	3.14±1.74	4.68±3.89	720.5	.160
Positive (≤6), 68.2%								
Neutral (7–13), 29.5%								
Negative (≥14), 2.3%								
Deliberation	0	21.0	0–24	6.90±4.28	4.65±2.35	7.21±4.76	611.5	.019
Sufficiently (≤12), 86.8%								
Uptake								
Accepted^†^, 75.2%								
Declined, 24.8%								
Decisional conflict	0	68.75	0–100	26.88±12.96	20.76±8.45	25.61±13.40	730.5	.192
≤37.4, 81.4%								
≥37.5, 18.6%								
Uncertainty	0	83.33	0–100	30.49±15.68	25.53±12.15	27.98±18.46	854.0	.806
Informed	0	58.33	0–100	24.94±13.16	18.78±8.98	25.00±13.98	699.0	.093
Values clarity	0	75.00	0–100	27.00±15.02	20.50±11.96	25.30±16.43	777.5	.350
Support	0	66.67	0–100	25.71±16.10	19.58±10.91	22.92±14.28	773.5	.334
Effective decision	0	81.25	0–100	26.41±14.58	19.74±8.78	26.56±15.74	602.5	.013

MMIC: Multidimensional measure of informed choice; NIPT: noninvasive prenatal testing. Participants who had a neutral attitude were not included in the informed choice calculation.

† Those who underwent NIPT were counted as having accepted.

‡ Mann-Whitney U-test.

**Table 4.** Types of informed choice and uninformed choice (N=91)

Choice	Knowledge	Deliberation	Attitude	Uptake	n (%)
Informed (n=63)	Good	Yes	Positive	Yes	63 (69.2)
	Good	Yes	Negative	No	0 (0)
Uninformed (n=28)	Poor	Yes	Positive	Yes	14 (15.4)
	Poor	Yes	Positive^†^	No^†^	3 (3.3)
	Poor	No	Negative	No	2 (2.2)
	Poor	Yes	Negative^†^	Yes^†^	1 (1.1)
	Poor	No	Positive^†^	No^†^	1(1.1)
	Good	Yes	Positive^†^	No^†^	6 (6.6)
	Good	No	Positive	Yes	1(1.1)
	Good	No	Negative	Yes	0 (0)
	Poor	No	Positive	Yes	0 (0)
	Poor	Yes	Negative	No	0 (0)
	Good	No	Negative	No	0 (0)
	Good	No	Positive	No	0 (0)
	Good	Yes	Negative	Yes	0 (0)
	Poor	No	Negative	Yes	0 (0)

† Value inconsistencies regarding noninvasive prenatal testing.

**Table 5.** Logistic regression for predicting informed choice (N=91)

Variable	Categories	OR (95% CI)	
		Univariate	Multiple
Age (year)	≤34	1	1
	≥35	0.62 (0.25–1.53)	0.42 (0.03–5.86)
Education level	≤College	1	1
	≥University	2.46 (0.57–10.64)	2.25 (0.15–34.97)
Financial status	High/middle-high	0.95 (0.38–2.37)	0.89 (0.15–5.43)
	Middle/middle-low	1	1
Job	No	1	1
	Yes	0.94 (0.38–2.31)	0.38 (0.04–3.59)
Religion	No	1	1
	Yes	1.21 (0.47–3.12)	1.39 (0.18–11.02)
Individual experience with people with chromosomal disability	No	1	1
	Yes	2.04 (0.62–6.79)	2.02 (0.19–21.39)
Parity	Nulliparous	1	1
	Parous	0.72 (0.28–1.86)	0.25 (0.03–2.26)
Reason for introducing NIPT	Wanted	1	1
	Reasons related to a high-risk pregnancy	0.34 (0.14–0.86)	0.57 (0.06–5.11)
Place of prenatal care	Tertiary general hospital	3.64 (1.05–12.55)	15.25 (0.83–281.04)
	Women’s hospital	2.12 (0.71–6.32)	9.16 (0.97–86.42)
	Obstetrical/gynecologic clinic	1	1
Knowledge		3.38 (2.02–5.66)	4.77 (2.17–10.47)
Attitude		0.80 (0.66–0.97)	0.89 (0.47–1.66)
Deliberation		0.79 (0.67–0.93)	0.74 (0.57–0.98)
Decisional conflict		0.95 (0.91–1.00)	1.08 (0.98–1.20)

CI, Confidence interval; NIPT: noninvasive prenatal testing; OR, odds ratio. Model χ2(14)=63.35 (*p*<.001), Hosmer-Lemeshow (*p*=.753), Cox and Snell R^2^=.501, Nagelkerke R^2^=.707.
